# The ALBS (Albert-Lembert with Knotless Barbed Sutures) Method for Esophagojejunostomy After Totally Robotic Radical Total Gastrectomy

**DOI:** 10.1007/s11605-023-05826-2

**Published:** 2023-09-06

**Authors:** Zongheng Wang, Shuangyi Ren, Bo Wang

**Affiliations:** Department of Gastrointestinal Surgery, The Second Affiliated Hospital of Dalian Medical University, 467 Zhongshan Road, Dalian, Liaoning 116023 People’s Republic of China

## Introduction

With the application of minimal invasive surgery during radical total gastrectomy, hand-sewing which is technology dependent is restricted. However, the utilization of robotic system has changed this situation. It features multi-articulated instruments with six degrees of freedom, which makes it more flexible compared to laparoscopic surgery. It also provides intuitive motion of the surgical instrumentation and tremor reduction with motion scaling.^1^ These unique advantages make hand-sewn esophagojejunostomy easier and more feasible. Therefore, our center applied the Albert-Lembert with knotless barbed sutures (ALBS) method for esophagojejunostomy during totally robotic total radical gastrectomy.

## Materials and Methods

### Patients

From December 2019 to August 2022, 30 patients underwent totally robotic radical total gastrectomy with the ALBS method for esophagojejunostomy by one experienced surgeon (Shuangyi Ren). All patients signed the written informed consent.

### Surgical Procedure

**Fig. 1:**
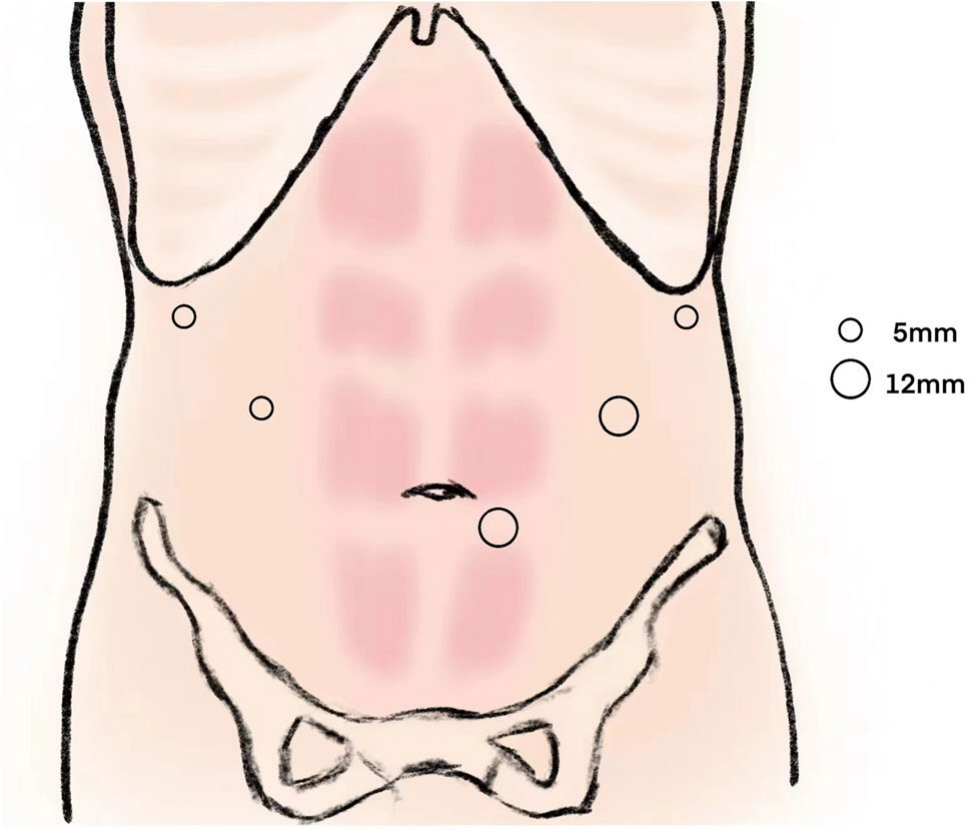
The positions of the trocars

With general anesthesia, the positions of the trocars are as shown in Fig. 1.

**Fig. 2:**
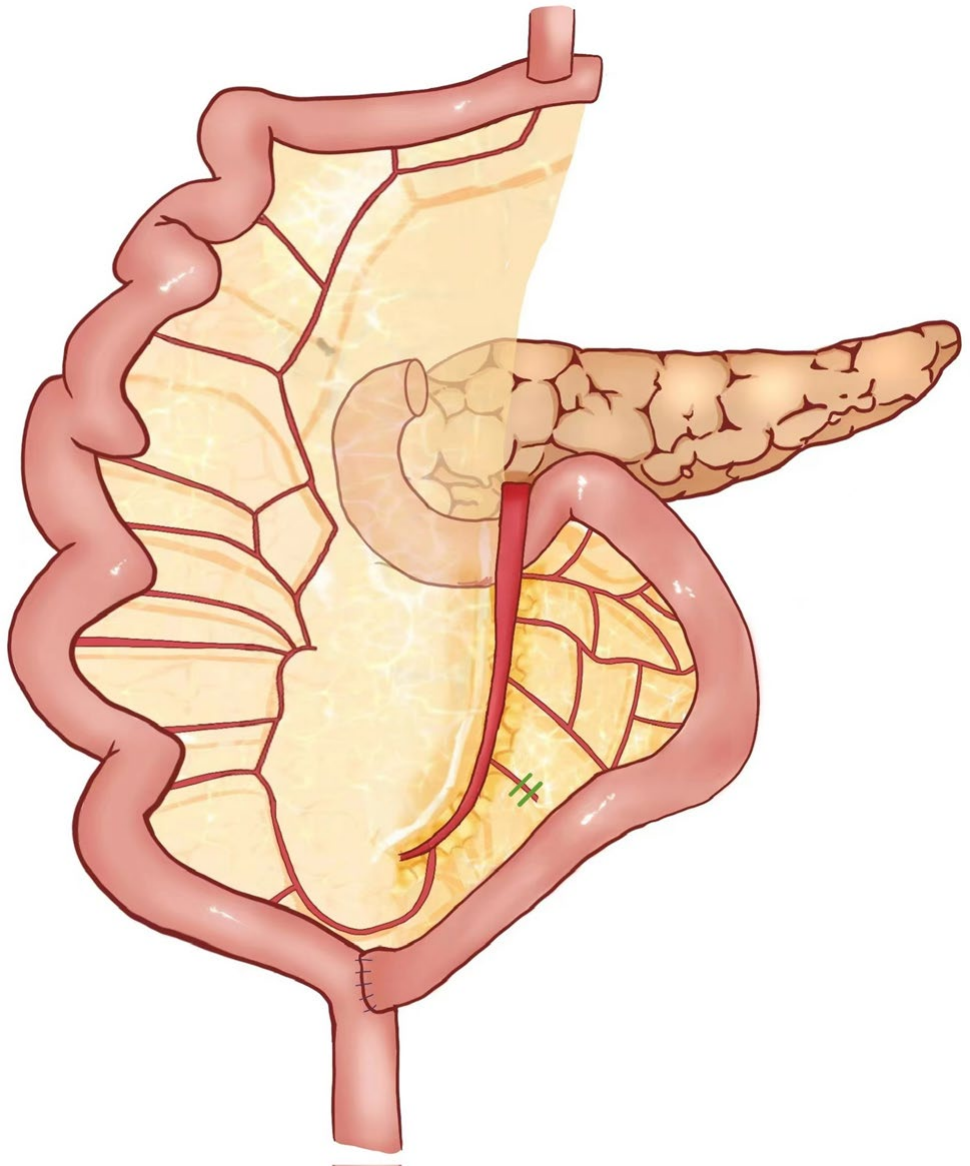
The preparation of the jejunum

Firstly, we completed total gastrectomy and D2 dissection which involved the resection of No. 1–7, 8a, 9, 11, and 12a lymph nodes.^2^ We transected the distal esophagus using a liner stapler with the proximal margin 2 cm approximately. Then we transected the jejunum at the 20 cm away from the Treitz ligament, and the Roux limb was prepared as shown in Fig. 2.

**Fig. 3:**
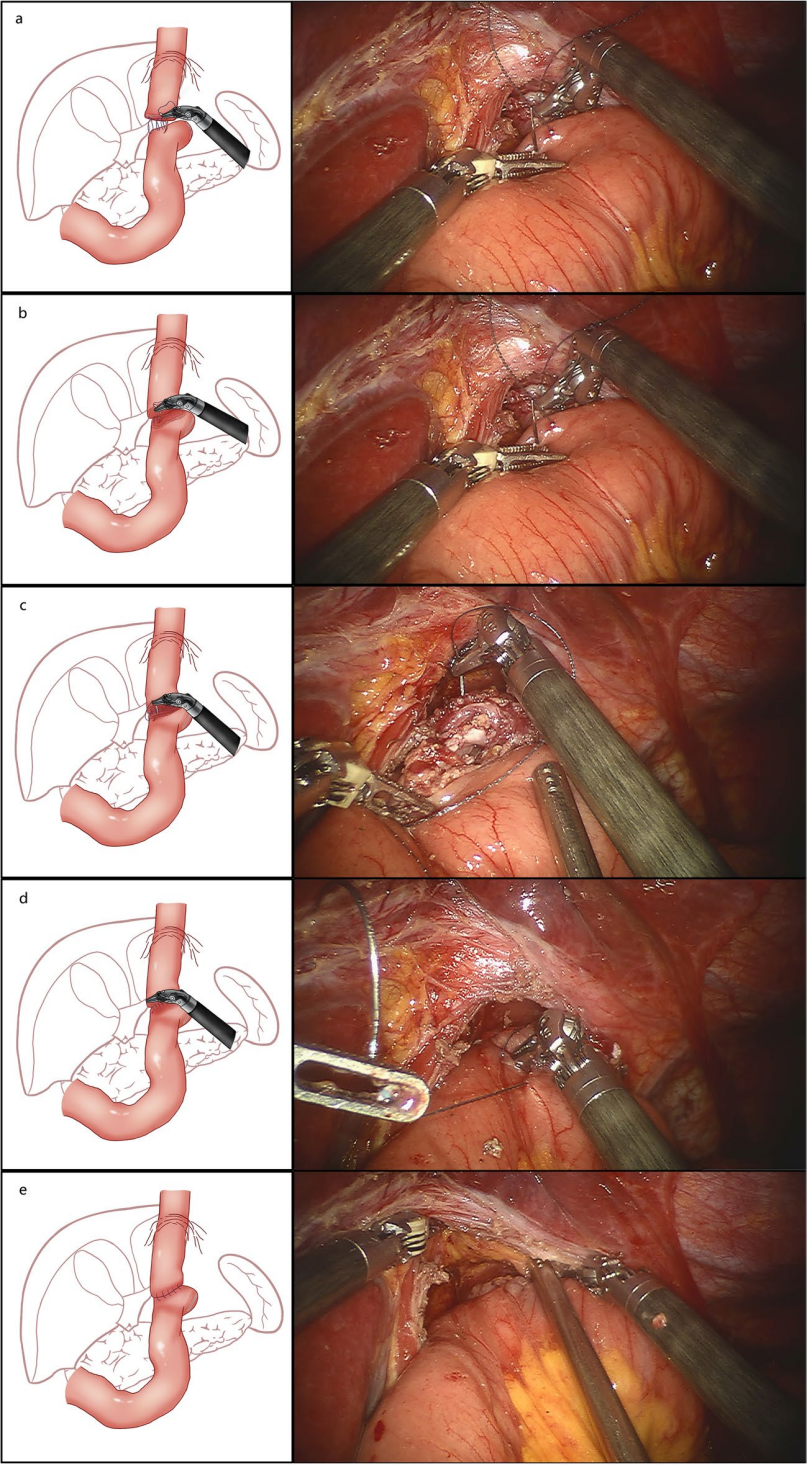
The specific procedure of the ALBS method

Secondly, we started the esophagojejunostomy with the ALBS method. It needs three 15-cm 3-0 knotless barbed sutures. Figure 3 shows the procedure.

- The surgeon sutured the posterior wall of the esophagus and jejunum in the seromuscular layer from the left to right. The suture distance is about 0.5–1 cm, and the stitch distance is about 1 cm. In this step, we completed the first layer of suturing for this method, simultaneously providing better stabilization of the esophagus, which simplifies subsequent suturing and prevents excessive tension that may lead to damage to the esophagus.
- The stump of the esophageal was cut by the harmonic scalpel and the anterior wall of the jejunum was opened about 2 cm. Pay attention to the neatness of the cutting margin to avoid the formation of pseudo-fornix between the mucosa and submucosa.
- The posterior wall of the esophagus and jejunum was sutured in full thickness while being careful not to miss the mucosal layer. It was crucial to adhere to the suture distance of the seromuscular layer and maintain a uniform stitch distance.
- The anterior wall of esophagus and jejunum was sutured in full thickness. Avoid to suture the posterior wall of anastomotic site, and ensure proper inversion of the jejunum’s edge.
- The anterior wall of the esophagus and the anterior wall of jejunum was continuously sutured using the seromuscular layer. Do not invert excessively to prevent anastomotic stenosis.

Finally, we performed a side-to-side anastomosis of the proximal and distal jejunum. Then we extend the observation trocar and removed the specimen.

## Results

**Fig. 4:**
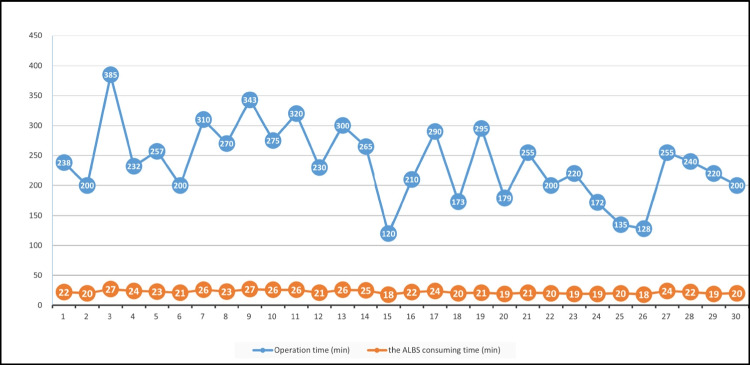
Times taken in each of the 30 patients to perform the total operation and the ALBS consuming time which was defined as the time from the barbed suture entering the jejunal stump to the end of anastomosis

**Fig. 5:**
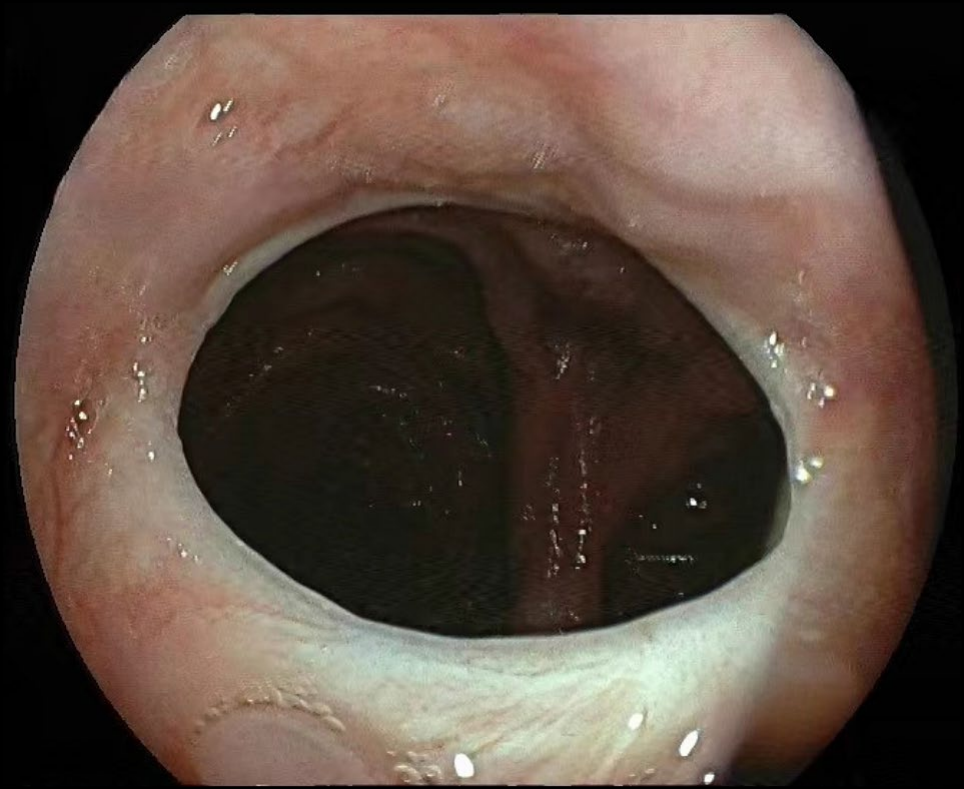
Endoscopic view of the esophagojejunostomy at postoperative 6 months after the ALBS procedure

A total of 30 patients underwent esophagojejunostomy using the ALBS method. Table 1 provides a summary of their clinical characteristics, surgical features, and outcomes. The total operation times and the ALBS consuming time of these 30 patients are shown in Fig. 4. The follow-up endoscopy was conducted at postoperative 6 months (Fig. 5).

**Table 1:** Clinical characteristics, surgical features, and complications of the patients

	*n *= 30
Age (years)	64.3 ± 13.1
Sex (M/F)	24:6
Body mass index (kg/m^2^)	23.7 ± 3.1
Previous abdominal surgery	8 (26.7%)
Tumor maximal size (cm)	5.0 ± 2.5
Operation time (min)	237.2 ± 62.8
The ALBS consuming time (min)	22.1 ± 2.8
Estimated blood loss (ml)	94.6 ± 76.6
Tumor infiltration depth	
T1	3 (10.0%)
T2	5 (16.7%)
T3	9 (30.0%)
T4	13 (43.3%)
Node metastasis	
0 (N0)	5 (16.7%)
1–2 (N1)	12 (40.0%)
3–6 (N2)	3 (10.0%)
≥7 (N3)	8 (26.7%)
Anastomotic bleeding	0
Anastomotic leakage	1 (3.3%)
Anastomotic stenosis	0
Postoperative hospital stays (days)	11.3 ± 5.1

## Discussion

Because of the challenges of performing hand-sewn laparoscopically, esophagojejunostomy is primarily conducted using linear or circular staplers. However, staplers cannot avoid the problems associated with anvil implantation and anastomotic stenosis.^3^ With the development of robotic surgery, hand-sewing in minimally invasive surgery has become easier and more feasible.

Our study demonstrates that the ALBS method is both safe and feasible in terms of time and complications for esophagojejunostomy during totally robotic total radical gastrectomy. The average operative time was 237.2 min with a mean ALBS consuming time of 22.8 min. Although the operation time varied significantly among different patients, the ALBS consuming times were relatively stable. During the postoperative recovery period, none of the 30 patients experienced anastomotic bleeding. Only one patient had mild anastomotic leakage, who recovered after parenteral nutrition for 5 days. This maybe because he was elderly and severely malnourished before the surgery. Regarding long-term anastomotic complications, none of the 30 patients developed anastomotic stenosis.

Compared to stapler anastomosis, hand-sewn anastomosis during robotic surgery offers several advantages. Firstly, under the condition of the same proximal margin, it can save more of the esophagus which is beneficial to the resection of high-seated tumors, especial for Siewert II. Secondly, hand-sewn anastomosis does not require a long-distance esophageal stump, which means the end of the esophagus can get more blood supply. Thirdly, the sutures are absorbable, which can reduce the occurrence of esophageal strictures theoretically. In addition, it can save costs compared with stapler method.

However, our findings still have limitations. It was a single-center study and the number of patients in the study population was relatively small. More surgeries will be performed to further verify this conclusion.

## Conclusion

Our results suggest that the ALBS is a safe and feasible method for esophagojejunostomy during totally robotic total radical gastrectomy.

## Declarations

### Confidentiality and Privacy

The privacy and confidentiality of the participants’ personal and medical information were strictly maintained throughout the study. All data were anonymized and coded to prevent the identification of individual participants.
